# Fibroblast-Derived Extracellular Matrices: An Alternative Cell Culture System That Increases Metastatic Cellular Properties

**DOI:** 10.1371/journal.pone.0138065

**Published:** 2015-09-15

**Authors:** Michael T. Scherzer, Sabine Waigel, Howard Donninger, Vennila Arumugam, Wolfgang Zacharias, Geoffrey Clark, Leah J. Siskind, Patricia Soucy, Levi Beverly

**Affiliations:** 1 J. G. Brown Cancer Center, University of Louisville, Louisville, Kentucky, 40202, United States of America; 2 Department of Bioengineering, University of Louisville, Louisville, Kentucky, 40202, United States of America; 3 Department of Medicine, University of Louisville, Louisville, Kentucky, 40202, United States of America; 4 Department of Pharmacology and Toxicology, University of Louisville, Louisville, Kentucky, 40202, United States of America; University of Kentucky College of Medicine, UNITED STATES

## Abstract

Poor survival rates from lung cancer can largely be attributed to metastatic cells that invade and spread throughout the body. The tumor microenvironment (TME) is composed of multiple cell types, as well as non-cellular components. The TME plays a critical role in the development of metastatic cancers by providing migratory cues and changing the properties of the tumor cells. The Extracellular Matrix (ECM), a main component of the TME, has been shown to change composition during tumor progression, contributing to cancer cell invasion and survival away from the primary cancer site. Although the ECM is well-known to influence the fate of tumor progression, little is known about the molecular mechanisms that are affected by the cancer cell-ECM interactions. It is imperative that these mechanisms are elucidated in order to properly understand and prevent lung cancer dissemination. However, common *in vitro* studies do not incorporate these interactions into everyday cell culture assays. We have adopted a model that examines decellularized human fibroblast-derived ECM as a 3-dimensional substrate for growth of lung adenocarcinoma cell lines. Here, we have characterized the effect of fibroblast-derived matrices on the properties of various lung-derived epithelial cell lines, including cancerous and non-transformed cells. This work highlights the significance of the cell-ECM interaction and its requirement for incorporation into *in vitro* experiments. Implementation of a fibroblast-derived ECM as an *in vitro* technique will provide researchers with an important factor to manipulate to better recreate and study the TME.

## Introduction

The five-year survival rate for stage 3 lung cancer patients is only around 15% [1]. This poor survival rate is largely contributed to the metastatic form of the disease, which allows the cancer to become a systemic burden, by infiltrating vital organs. Approximately, 50% of patients with non-small-cell lung cancers (NSCLC), which is the classification for nearly 80% of all lung cancers, have metastatic lung cancer at diagnosis [2]. Although survival rates improve with early detection, there is a great need for efficacious therapies that treat the metastatic form of lung cancer. There are many FDA approved therapies that are successful for lung cancer patients (eg. surgical resection, local radiation, and chemotherapeutics), but few therapies exist that are effective at specifically targeting cancer cells, while leaving healthy cells untouched, and even fewer that are effective against the metastatic cancers. This failure to produce effective therapies is partly due to false discoveries that are attributed to a lack of appropriate in vitro models to accurately recapitulate the *in vivo* mechanisms that drive lung cancer and its progression to metastasis [3]. For instance, many cancer therapies are developed from chemicals that illicit a cancer specific cytotoxic response during *in vitro* cell culture environments, but these cell culture environments do not offer the full biological repertoire that is present within the tumor in a patient. Thus, researchers are limited in the accuracy of their conclusions, which leads them down an incorrect path that may ultimately result in failure in the clinical setting. Although cell culture experiments are a simple first-line test for new therapies, an improved *in vitro* model could filter out inefficacious treatments before large financial and temporal investments are made.

The extracellular matrix (ECM), an essential constituent of the tumor microenvironment (TME), is a meshwork of protein fibers and glycosanimoglycans (GAGs) that not only provides mechanical support, but also offers growth and migration cues through growth factors, adhesion interactions, and mechano-transduction [4]. The ECM is generally secreted and organized by fibroblasts, but other cells can contribute to ECM production, such as endothelial and epithelial cells [5]. Lately, the ECM has been heavily researched for its role in the progression of lung and breast carcinomas [5, 6]. The balance of ECM deposition and ECM degradation can potentiate diseases such as fibrosis and cancer [7]. Increased production of the high elastic modulus collagen and decreased low elastic modulus elastin expression can stiffen local tissue, therefore altering mechano-transduction pathways [8]. Matrix metalloproteases (MMPs) are matrix-degrading enzymes that can degrade the ECM and alter its elasticity, which can provide cells with important biomechanical stimulation to direct invasion into surrounding tissue and blood vessels, leading to metastasis [9]. Alternately, ECM can be stiffened by increased matrix production and deposition of collagen via Lysyl Oxidase (LOX) signaling [10]. For instance, ECM accumulation by increased collagen deposition has been documented in many tumor cell types, including glioma, breast, and lung cancers [11, 12]. This abnormal ECM can cause changes in the mechano-transduction pathways that regulate cell growth and migration pathways. Tension-induced signaling has been shown to affect Mitogen-Activated Protein Kinase (MAPK) signaling pathways by p44/42 activation in fetal lung epithelial cell lines [13]. MAPK signaling is highly affected in cancer that activates many downstream applications involved in cell growth and survival. Similarly, focal adhesions are the point of cell-ECM matrix interaction and are comprised of integrins that cluster together and bind the ECM, thus triggering downstream pathways mediated through Focal Adhesion Kinase (FAK) [6]. These downstream signaling pathways have the ability to modulate MMP and tissue inhibitors of metalloproteases (TIMP) that can adjust ECM synthesis and degradation [6]. It is now evident that there exists a complex feedback mechanism between cancer cells and ECM that influences the fate of the tumor [14]. Interference of the cancer-promoting ECM-cell interactions could immobilize cancer cells and inhibit the deadly metastatic form of lung cancer, thus improving patient survival rates. Therefore, more basic research is needed to understand how lung ECM affects lung cancer cells, and vice versa.

De-cellularized fibroblast-derived ECM could be a simple, cost-effective cell culture technique that recapitulates the ECM-lung cancer cell interactions that have been shown to play a vital role in the progression of cancer. Fibroblasts are the principal contributors to ECM synthesis in most types of tissue, including the lung. Therefore, an ECM synthesized by fibroblast that retains its structural architecture will more accurately represent the native tumor environment. Cukierman *et al* have published numerous articles describing techniques to harvest mouse fibroblast-derived matrices, and even characterized how these matrices affect the morphology, growth, and drug response of numerous epithelial tumors cell lines, but lung adenocarcinoma cells seeded onto human lung fibroblast-derived matrix were tested [15]. Further, the same group demonstrated that overexpression of Fibroblast-activated Protein (FAP) in 3T3 cells induced matrix fiber alignment, which increased the growth invasiveness of pancreatic cancer cells [16]. Still, little is known about how human lung fibroblast-derived matrices alter the morphology, growth, invasiveness, and biochemical properties of lung cancer cell lines.

In this work, we sought to better understand the effect of tissue-specific, human lung fibroblast-derived matrices on two lung adenocarcinoma cell lines, A549 and H358 and immortalized, but not transformed, HPL1D cells. Specifically, cell proliferation, microscopy, western blot, RNA analysis, and migration studies were used to characterize the affects of human fibroblast-derived ECM lung epithelial-derived cell lines.

## Methods

### Cell Culture

Three human fibroblast cell lines were used in this study, IMR90, WI38, and HDF, were all purchased from ATCC. IMR90 and HDF cells were both cultured in RPMI 1640 (Hyclone SH30027.01) supplemented with 10% FBS (Hyclone SH30070.03), 1% penicillin-streptomycin (Hyclone SV30030), and 1% L-glutamine (Hyclone SH30034.01). WI38 cells were cultured in MEM media (Gibco 10370–021) supplemented with 10% FBS, 1% penicillin-streptomycin, and 1% L-Glutamine.

Three-epithelial cell lines, A549, H358, and HPL1D were used to seed onto fibroblast-derived matrix were purchased from ATCC. HPL1D cells are immortalized peripheral lung epithelial cells and were used to compare the cancerous phenotype of A549 and H358 cell lines to normal. However, HPL1D cells have been shown to form soft-agar colonies in our lab, suggesting they are transformed. All epithelial cells were cultured in RPMI 1640 supplemented with 10% FBS, 1% Glutamine, and 1% penicillin-streptomycin.

To mimic hypoxia, cells were treated with 1mM cobalt chloride for 16 hours.

### Extracellular Matrix Production and Decellularization

ECM production and harvesting protocols were adapted from Cukierman *et al* and Soucy *et al* [17, 18]. Briefly, tissue culture treated dishes were coated with 50 μg/ml fibronectin (FN) in PBS for 1 hour at 37 degrees Celsius. Excess FN was removed and fibroblasts are seeded at confluence, and cultured for 8 days while changing the media every two days. HDF cells are a little larger that the lung fibroblast cell lines so they are seeded at a lower density than WI38 and IMR90 cells. For example, in a 6 well dish, 300,000 IMR90 or WI38 cells are seeded, but for the same size dish, 270,000 HDF cells were seeded. After 8 days of culture, cells are washed once with PBS (Hyclone SH30028.02), and then incubated at room temperature for 2 minutes in PBS with 50 mM ammonium hydroxide (NH_4_OH) (Fischer-A669-500) with 0.05% Triton X-100 (Sigma T8787). Cells are constantly observed with a light microscope at 10X to confirm proper removal of all fibroblast debris. After decellularization, the matrices are washed 3 times with PBS. To prevent unwanted fibroblast DNA, which could affect downstream applications, matrices are incubated at for one hour at 37°C in a 20U/mL DNAse 1(Thermo Scientific EN0525) in sterile H_2_O (CellGro 25-055-CV). After DNAse 1 treatment, matrices are washed 3 times with PBS and either stored at 4°C for up to one week, or used immediately.

### Microscopy and Fluorescent Microscopy

For light microscopy, a Zeiss AX10 inverted microscope was used at 10X and 40X. For Immunofluorescence, a Nikon A1R Spectral Confocal microscope was used. For immunofluorescence, matrices and/or cells were fixed with 4% formaldehyde in PBS for 20 minutes at room temperature then washed 3 times with PBS for 5 minutes each. For ECM staining, a succinimidyl ester (NHS ester) conjugated to an Alexa Fluor 488 dye was incubated at a concentration of 10 μg/mL for 30 minutes at 37°C, then washed 3 times with PBS for 5 minutes. To ensure no leftover fibroblast DNA is present, DAPI was also added at a concentration of 1:1000 in PBS for 10 minutes at room temperature. To image the cytoskeleton of epithelial cells, phalloidin was added at a concentration of 1:1000, while incubating with DAPI for 10 minutes at room temperature. After staining, the cells were imaged.

### SDS PAGE and Western Blot

Cells were lysed in CHAPS buffer (1% (w/v)CHAPS in 150 mM NaCl, 50 mM Tris pH 7.5, 50 mM EDTA) containing protease and phosphatase inhibitors for 20 minutes on ice. Lysates were centrifuged at 15,000 x g for 8 minutes at 4°C to pellet the insoluble material, which includes the extracellular matrix proteins. Protein concentration was then quantified using a bicinchoninic acid (BCA) assay (Thermo Scientific #23228, #1859078). Protein (30 μg) of total protein was loaded on a 4–12% bis-tris polyacrylamide gel (Novex) and 150 volts was applied across the gel for 60 minutes at room temperature while the gel was submerged in 1X running buffer (Novex NP0002) in water. After protein migration, gels were either stained with colloidal blue (Invitrogen LC6025) for 4 hours to stain total (w/v) milk in a mixture of Tris-buffered saline and Tween 20 (TBS-T) to block non-specific binding by the antibody. Primary antibodies specific to indicated proteins were acquired from Cell Signaling™ and were added at a concentration of 1:10000 in 5% milk and incubated with the membranes overnight at 4°C. After primary antibody incubation, membranes were washed 3 times in 5% milk for 5 minutes. Secondary antibody, conjugated to HRP, specific to target the primary rabbit antibody, was incubated with the membranes in 1.25% (w/v) milk for 30 minutes at room temperature then washed twice for 5 minutes each in 1.25% (w/v) milk. Membranes were then incubated for 20 minutes in TBS-T before adding HRP substrate (Thermo Scientific #34095) for detection by luminescence captured on a photographic film.

### Cell Growth

To determine the effect of cell-derived matrices on cell growth and proliferation, two assays were employed. To determine cell number, 30,000 cells were plated in a 12 well dish with a fibronectin coat, or fibroblast-derived ECM. Every 24 hours for 4 days, cells were washed in PBS, trypsinized (Hyclone SH30042.01) for 3 minutes at 37°C, centrifuged at 1200 RPM for 3 minutes and suspended in 1 mL media for counting with Trypan Blue™ (Invitrogen T10282) in a Countess automated cell counter (Invitrogen). Alternately, 2,000 cells were seeded into a 96 well-plate, and Alamar Blue™ (Invitrogen 612130) was added each day for 4 days to determine relative cell numbers. Raw fluorescent data were averaged, with the blank reading subtracted, and then normalized to control plates.

### Trans-well Migration Assay

To determine if fibroblast-derived matrices affected how epithelial cells migrate, trans-well chamber (Fischer 353097) assays were used. Briefly, ECM was produced on the bottom side of a trans-well chamber and A549 cells were cultured on the top side. After 24 and 48 hours, non-migrating cells were scraped off the top of the membrane. Migrating cells on the bottom of the membrane were then fixed in 4% paraformaldehyde and stained with hemotoxylin and eosin (Protocol 122–911). Membranes were then mounted on a glass slide and migrated cells were counted using ImageJ software analysis. n = 4.

### Microarray

After 48 hours on ECM or fibronectin (FN), RNA was harvested using E.Z.N.A Total RNA Kit (Omega) and analyzed on a nanodrop 1000 (Thermo Scientific) and Bioanalyzer 2100 (Agilent Technologies) for quality control. RNA samples were then amplified converted to cDNA, labeled using 3’ IVT Express Labeling Kit (#901229), and hybridized on an Affymetrix PrimeView Human Array Chip. The chip was scanned on an Affymetrix GeneChp 7G scanner. Raw intensity scores were imported into Partek Genomics Suite 6.6 (6.13.0731) and normalized on a gene level using the standard Robust Multi-array Average (RMA) algorithm for normalization and background correction. A two-way ANOVA was set up and a step-up FDR corrected P-value was included for every P-value calculated. Only significant gene changes with a P-value of ≤0.05 and a fold change greater than 1.5 were uploaded into MetaCore™ for pathway and gene otology analysis.

### Real Time PCR

New biological replicates were used to generate RNA as previously described for the microarray. Complementary DNA was generated using the RNA to cDNA kit (Applied Biosystems). This kit uses thymidine-rich primers that bind to the poly-A tail of mRNA and allow a polymerase to synthesize the complete complimentary strand. Oligonucleotide sequences are in Table 1.

**Table 1:** 

Gene	Sense	Antisense
LGALS	GGTCAACCCTGAAGATCACAG	GTCCAATGAGTTGCAGACAATG
PSAT1	AAGGTGTGCTGACTATGTGG	TTGAGGTTCCAGGTGCTTG
ASNS	AGGAGAGTGAGAGGCTTCTG	GGTGGCAGAGACAAGTAATAGG
BCAT1	AATCCCAAGTATGTAAGAGCCTG	AAGAGATGAGCCGTAATTCCC
IL-8	ATACTCCAAACCTTTCCACCC	TCTGCACCCAGTTTTCCTTG
STAT4	CCTGAAAACCCTCTGAAGTACC	ACCTTTGTCACCCCTTTCTG
C3	AACTACATCACAGAGCTGCG	AAGTCCTCAACGTTCCACAG
C1S	TTTGTAGATGTCCCTTGTAGCC	AATCTCCCCAATCAGTGCAG
MMP7	TTCCAAAGTGGTCACCTACAG	AGTTCCCCATACAACTTTCCTG
RND1	ATGTAAGCTCGTTCTGGTCG	CTCTGTTCCTCTGTCTCCAAAC
GAPDH	TGCACCACCAACTGCTTAGC	GGCATGGACTGTGGTCATGAG

Primers were designed to be between 18–22 base pairs long, have a 40%-60% GC content, and amplify a product between 60–120 base-pairs. Primer specificity was validated to only amplify one product at 60°C. Reaction efficiency was also validated to ensure that fluorescence intensity doubled each cycle using a serial dilution of a known cDNA template. Reactions were shown to be efficient between 10 ng and 50 ng concentrations of DNA. To qualify, efficiency must be within 85% and 105% with an R^2^ value greater than 0.98. This quality control confirms that no matter the differences in original starting material between the wells, comparison of threshold values are valid because the samples are within the linear range. To calculate relative fold change compared to a fibronectin-coated dish, the 2 ^ΔΔC^_T_ method was employed using the following equation:

ΔΔCT=(ΔCT(ECM)−ΔCT(FN))

ΔCT=Average CT (Target gene)−Average CT (GAPDH).

C_T_ is the PCR cycle number where the fluorescent threshold value is reached. All PCR reactions were analyzed on a Biorad CX96.

### Time-lapse microscopy

150,000 A549 cells were seeded onto either ECM-coated or fibronectin-coated 35mm dishes with a coverslip insert for microscopy. 24 hours after cell seeding, dishes were inserted into a Tokai Hit environmental chamber for 24 hours and pictures were taken at 3 different fields every 10 minutes, for 24 hours. Cell migration was calculated by tracking 4–8 cells per field throughout the time lapse. Cell tracking was achieved by the MtrackerJ plugin, available for ImageJ [19]. Average point by point velocity for all lines were calculated within MtrackerJ and reported with SEM. Directionality, a measure to determine linearity of migration, was calculated by D/d.

### Cell Circularity

After confocal microscopy with phallodin and DAPI staining, images with scale bars were imported in ImageJ and converted from RGB to 32-bit and a threshold was set to create maximum contrast between cell borders and empty space. Built-in functions of ImageJ allowed for easy image segmentation for area and perimeter analysis. Circularity is a value between 0–1 with 1 being a perfect circle [20] and is calculated using the following equation.

$$Circularity=4\pi \times \left(Area÷Perimeter^{2}\right)$$

### Statistical Analysis

A one-way ANOVA with a Tukey’s Test for multiple comparisons were used to determine statistical significance, unless otherwise stated. *, p-value ≤ 0.05

## Results and Discussion

**Fig 1:**
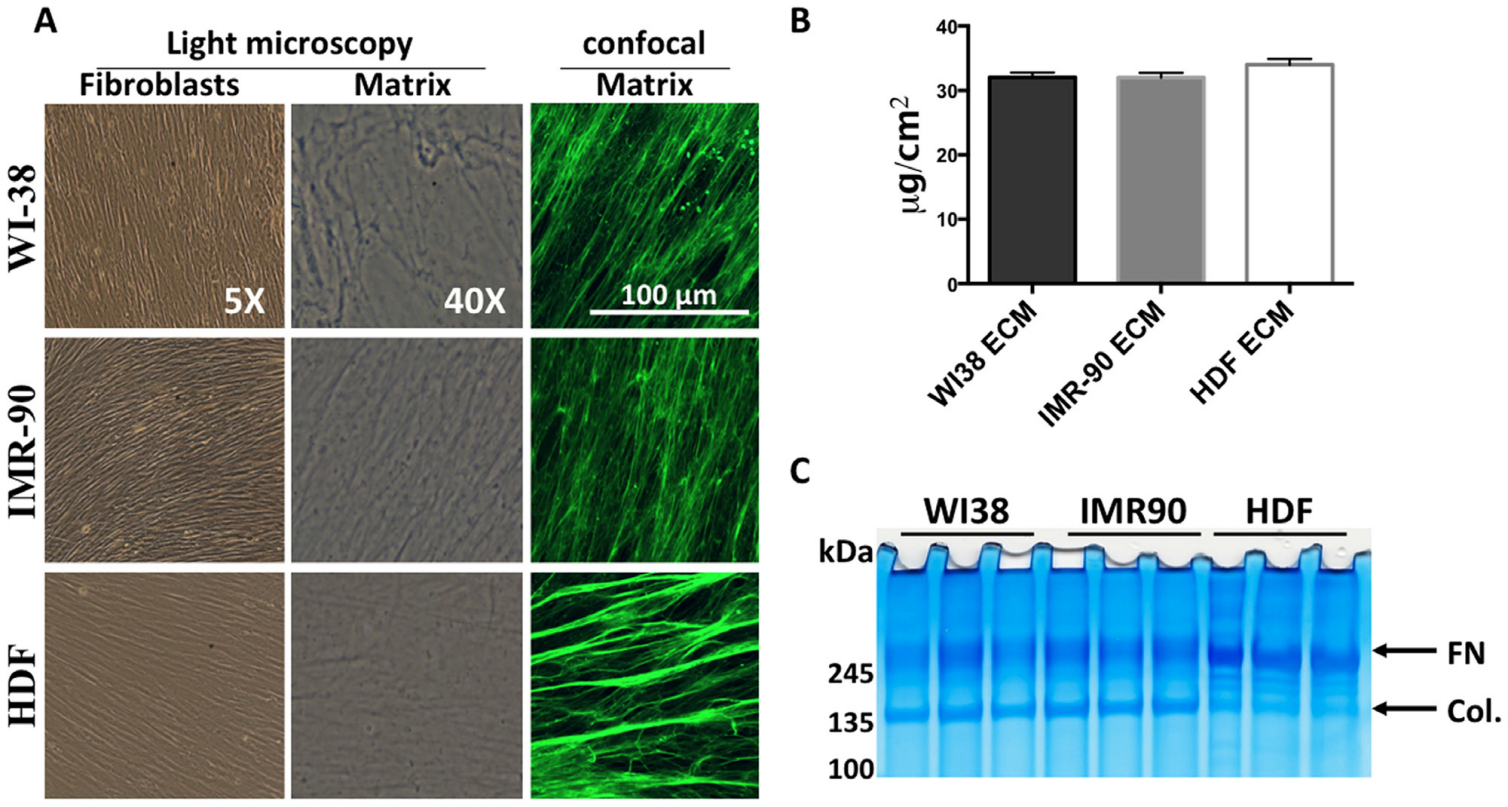
Characterization of fibroblasts and their ECM. A. Microscopic analysis of fibroblasts and de-cellularized matrix. Left colum- 5X phase contrast imaging of confluent fibroblasts just prior to de-cellularazation. Middle colum- 40X phase contrast microscopy of ECM following decellularization. Right column- Immunofluorescent confocal microscopy of fibroblast-derived ECM using an NHS-ester probe conjugated to Alexa Fluor 488. (B) Protein content of ECM was quantitated after scraping of the ECM from decellularized plates. Protein amount is indicated a μg/cm^2^, N = 3. (C) SDS-PAGE and colloidal blue stain of fibroblast-derived ECM. n = 3.

The goal of this work was to determine if fibrobast-derived ECM could alter cellular phenotypes of lung epithelial cell lines. To this end, we acquired two human fetal lung fibroblasts cell lines, WI38 and IMR90, and one human dermal fibroblast cell line, HDF. All fibroblast cell lines exhibited the same spindle-shaped morphological features (Fig 1A, left column). Interestingly, all three fibroblast cell lines aligned themselves in a linear pattern, and produced a similar ECM, with a similar highly linearized configuration (Fig 1A, center column and right column). This linearized ECM could be important for maximizing physiologic relevance to the human disease *in vitro*, which is not available with solubilized ECM, such as Matrigel. The resultant fibroblast-derived ECM was solubilized in 5M Guanidine-HCL and protein content was measured using a BCA assay (Fig 1B). All fibroblast-derived matrix produced similar amounts of ECM per cm^2^. WI38 ECM averaged 32.06 μg/cm^2^ ± 3.961, IMR90 ECM averaged 32.02 ± 3.299 μg/cm^2^, and HDF ECM averaged 34.016 ± 3.990 μg/cm^2^. Results were averaged from three wells of fibroblast-derived matrix solubilized in the same volume. After solubilizing, ECM protein was analyzed by SDS-PAGE and total protein was stained by colloidal blue (Fig 1C). Interestingly, both WI38 and IMR90 derived matrices have slightly more intense collagen bands (140 kDa- 175 kDa) whereas, HDF-derived ECM has a slightly more intense fibronectin band (260 kDa-280 kDa) compared to WI38 and IMR90 matrices. Collagen and fibronectin bands are easily identified because they have been previously shown to be the prominent constituent of WI38-derived ECM [18]. Also, there appears to be more intermediate protein bands that range between 175 kDa and 250 kDa that are more prominent in HDF derived ECM than WI38 and IMR90 ECM. However, it is not possible to conclusively determine the identity these proteins. It is interesting that there are differences in ECM protein expression between cell lines from two distinct tissue sources. These subtle differences in protein expression might alter the phenotype of lung cancer cell lines, thus potentially affecting the outcome of the human disease. These data indicate the tissue-specific alterations in ECM production and imply ECM may differentially regulate downstream signaling pathways through altered cell-ECM interactions.

### Fibroblast-derived Matrices Alter Lung Cancer Cell Morphology

**Fig 2:**
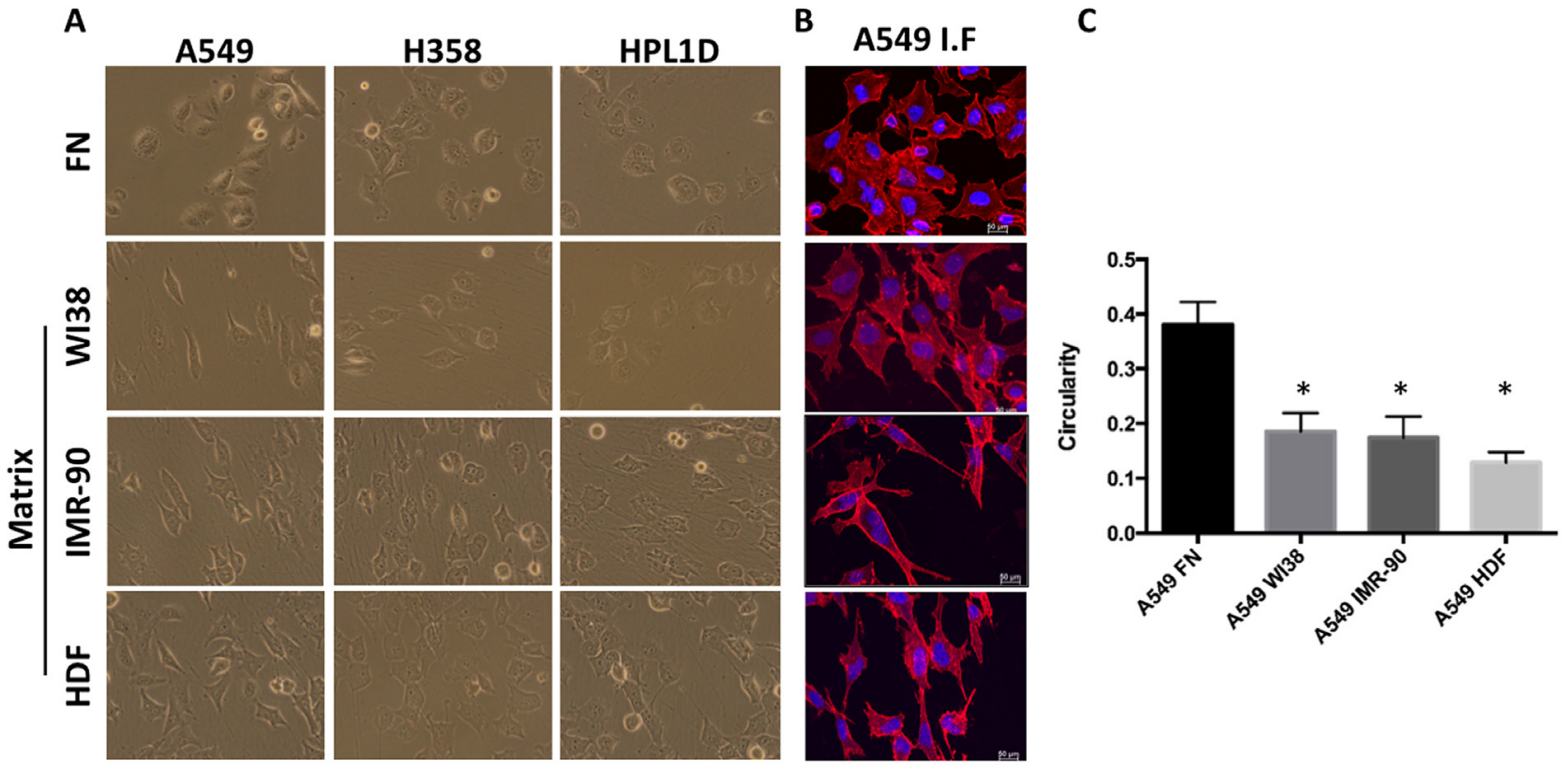
Fibroblast-derived ECM alter Lung Cancer Cell Line Morphology. (A) Phase contrast microscopy photos of A549, H358, and HPL1D cells on FN, WI38 ECM, IMR90, and HDF ECM. (B) Immunofluorescent confocal microscopy of A549 cells on WI38-derived ECM stained with Phallodin and DAPI. (C) The circularity of A549 on FN and 3 different fibroblast-derived ECM was calculated. n = 10. *, p ≤ 0.05.

Once fibroblast-derived matrices were produced, we wanted to determine if cell morphology was altered when lung epithelial cells were seeded on human lung-specific matrix, human dermal matrix, or a normal fibronectin—coated (FN) tissue culture treated dish. Fibronectin-coated dishes were used as a control for two reasons. First, the plates are pre-coated with FN before fibroblasts were seeding to aid in attachment of the matrix and second, it simulates both the traditional 2D cell culture environment as well as having a natural ECM component. It should be noted that we have compared FN coated dishes to non-coated tissue culture plates and have not seen any differences in any phenotypes (not shown). After fibroblast extraction and ECM purification, A549, H358, and HPL1D cell lines were seeded onto the fibroblast-derived matrices or FN-coated dish, and then imaged 48 hours later to determine morphology. Observation by phase-contrast microscopy (Fig 2A) revealed marked differences in cell shape. A549 cells exhibited a dramatic spindle shaped morphology, while H358 and HPL1D cells exhibited a slight spindle-shaped morphology. Phalloidin, a chemical that has a high specificity for filamentous actin, was used to visualize the cytoskeleton of the cell by confocal microscopy (Fig 2B). Simple geometric calculations were used to judge cell circularity (Fig 2C). All three fibroblast-derived matrices significantly (p 0.05, n = 10) pressured A549 cells to form a more elliptical shape (circularity = <1), rather than the more circular shape (circularity ~1) conferred by the A549 cells cultured on fibronectin (Fig 2C). A549 cells on fibronectin have an average circularity value of 0.38 ± 0.04, while the same cells on WI38, IMR90, and HDF ECM have average circularity values of 0.18 ± 0.034, 0.18 ± 0.038, and 0.12 ± 0.019, respectively (Fig 2C). Cells settled between matrix fibers and elongated according to the direction of the fibers. However, the type of ECM did not have differential effects on cell morphology, suggesting that differences in the type and content of the ECM does not induce morphological change in lung cancer cell lines.

Changes in cell morphology can induce drastic alterations in cell processes such as proliferation and migration, thus influencing the fate of the cancer cells. In fact, it has been previously reported that cell shape can influence the metastatic capabilities of some cancers [21]. Cell elongation may contribute to increased cell migration. It is interesting that ECM imposes a drastic change in morphology, suggesting that cells growing on this substrate may have altered growth and migration properties.

### Fibroblast-derived Matrices Alter Lung cancer Cell Growth

**Fig 3:**
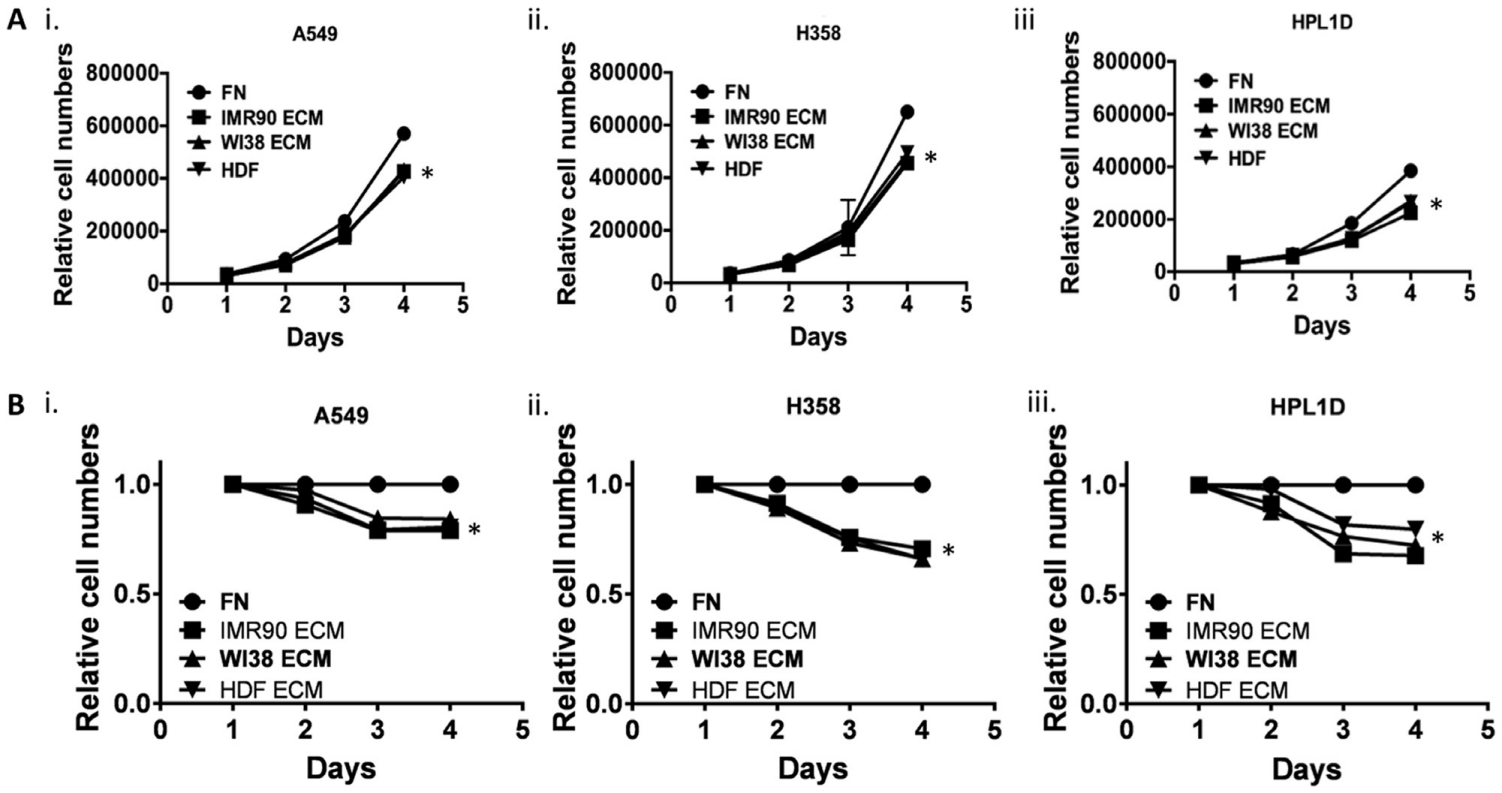
Fibroblast-derived ECM decreases Lung Cancer Cell Growth. (A) A549, H358 and HPL1D cells were grown on fibroblast derived matrices and every day one well of cells was trypsinized and manually counted in triplicate using trypan blue. (B) A549, H358 and HPL1D cells were grown on fibroblast derived matrices and every day Alamar Blue was added to wells of cells in triplicate and relative conversion of Alamar Blue was determined. n = 3. *, p-value ≤ 0.05.

Next, cell growth was monitored after lung epithelial cells were cultured on various fibroblast-derived matrices. We suspected that human fibroblast-derived matrices decreased cell growth because there seemed to be many fewer cells on ECM at the end of the culture periods. To test the hypothesis that lung cancer cells proliferate less when cultured on fibroblast-derived ECM, Alamar Blue™ and cell-counting assays were used. A549, H358, and HPL1D were seeded onto 12-well plates for Trypan blue™ cell counting, or 96-well plates for an Alamar Blue™ assay, and recorded measurements for four consecutive days (Fig 3A). It is clear that both cell counts (Fig 3A) and subsequent Alamar Blue metabolism (Fig 3B) were attenuated when cells were cultured on ECM. This result is consistent with previous work showing that various human epithelial cancers such as breast and colon cancer cell lines grow slower when cultured on mouse fibroblast-derived ECM [15]. It is the belief in the field that an increased extracellular matrix presence should enhance cancerous properties. However, this data suggests that fibroblast-derived ECM inhibit the growth of lung cancer cell lines. Nevertheless, more studies are needed that determine exactly how fibroblast-derived ECM slow down the growth of lung cancer cell lines, compared to the same cells on a fibronectin-coated tissue culture dish.

### Fibroblast-Derived Matrices alter functional protein levels of lung cancer cell lines

**Fig 4:**
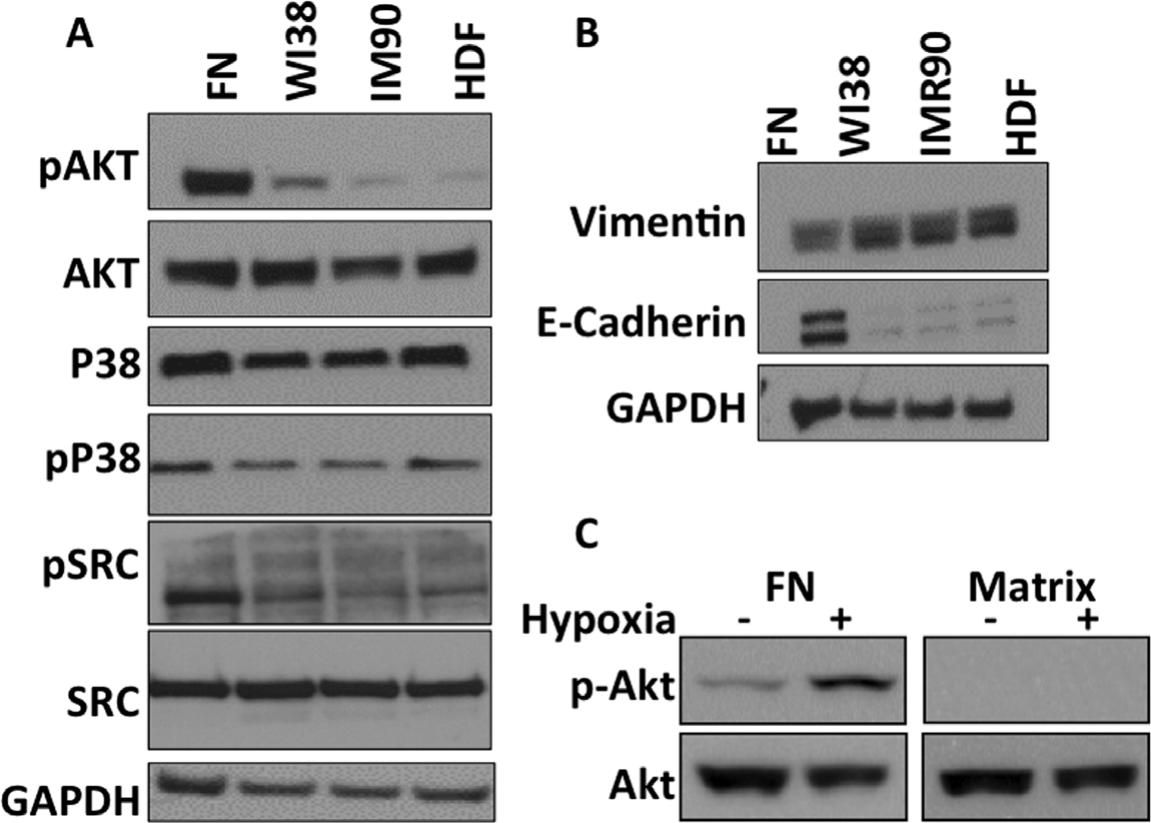
Fibroblast-derived ECM alters Protein Levels of A549 Cells. (A) A549 cells were cultured on the indicated substrate for 48 hours and then cells were harvested and western blots were performed with the indicated antibodies. (B) Cells were treated as in panel A and westerns were performed with indicated antibodies. (C) A549 cells were seeded on either fibronectin (FN) or WI38-derived ECM and then plates were incubated under hypoxic or normoxic conditions. Cells were harvested and westerns were performed with the indicated antibodies.

To begin to understand the growth-related signaling pathways that are altered when cells interact with fibroblast-derived ECM, western blot analysis was performed to detect various signaling proteins. Here, A549 cells cultured on FN, WI38 ECM, IMR90 ECM, and HDF ECM were lysed, followed by western blot analysis for proteins involved in MAPK signaling, Epithelial-to-Mesenchymal Transition (EMT) signaling, as well other proteins known to be involved in cellular proliferation. Interestingly, many proteins and phosphorylated proteins are decreased at the protein level, as detected by western blot (Fig 4). Phosphorylated MAPK family members such as P38 are decreased when A549 cells are cultured on all fibroblast-derived ECM (Fig 4A). MAPK initiation is known to activate many downstream pathways that affect cell cycle entry and cell growth. Similarly, AKT, SRC and Mammalian-Target of Rapamycin (MTOR), which are all together involved in several cascades that regulate cell cycle, are decreased when A549 cells are cultured on all fibroblast-derived matrices (Fig 4A). These data suggest possible effector molecules that are disrupted when A549 cells are cultured on fibroblast-derived ECM, thus providing key insight into the effect of the ECM on lung cancer cell line growth.

As previously stated, metastatic lung cancer is the deadliest and most aggressive form of the disease. EMT is a process initiated by cells in the primary tumor in order to migrate and become metastatic. Biomarkers of EMT are the loss of E-cadherin, and the gain on vimentin expression in epithelial cancers [22]. It is hypothesized that ECM can influence EMT biomarkers, thus inducing cells to undergo EMT. Interestingly, E-cadherin expression is greatly decreased and vimentin expression is increased when A549 cells are cultured on all fibroblast-derived matrices. These data suggest that fibroblast-derived ECM alter EMT markers of A549 cells, which could induce a more migratory phenotype, thus increasing likelihood of metastasis.

To demonstrate that our system can be combined with additional “more physiological” systems, we cultured cells on ECM under hypoxic conditions. This has the potential of modeling the most relevant conditions that cancer cells experience within the tumors. As previously reported, signaling pathways are altered by both ECM and hypoxia (Fig 4C). For example, on fibronectin hypoxia causes an increase in AKT signaling; however when cells are cultured on ECM, hypoxic conditions do not lead to increased AKT signaling. Further work will be necessary to elucidate the signaling pathways altered by these combined systems.

### Fibroblast Derived Matrices Protect Lung Cancer Cells from Serum Deprivation

**Fig 5:**
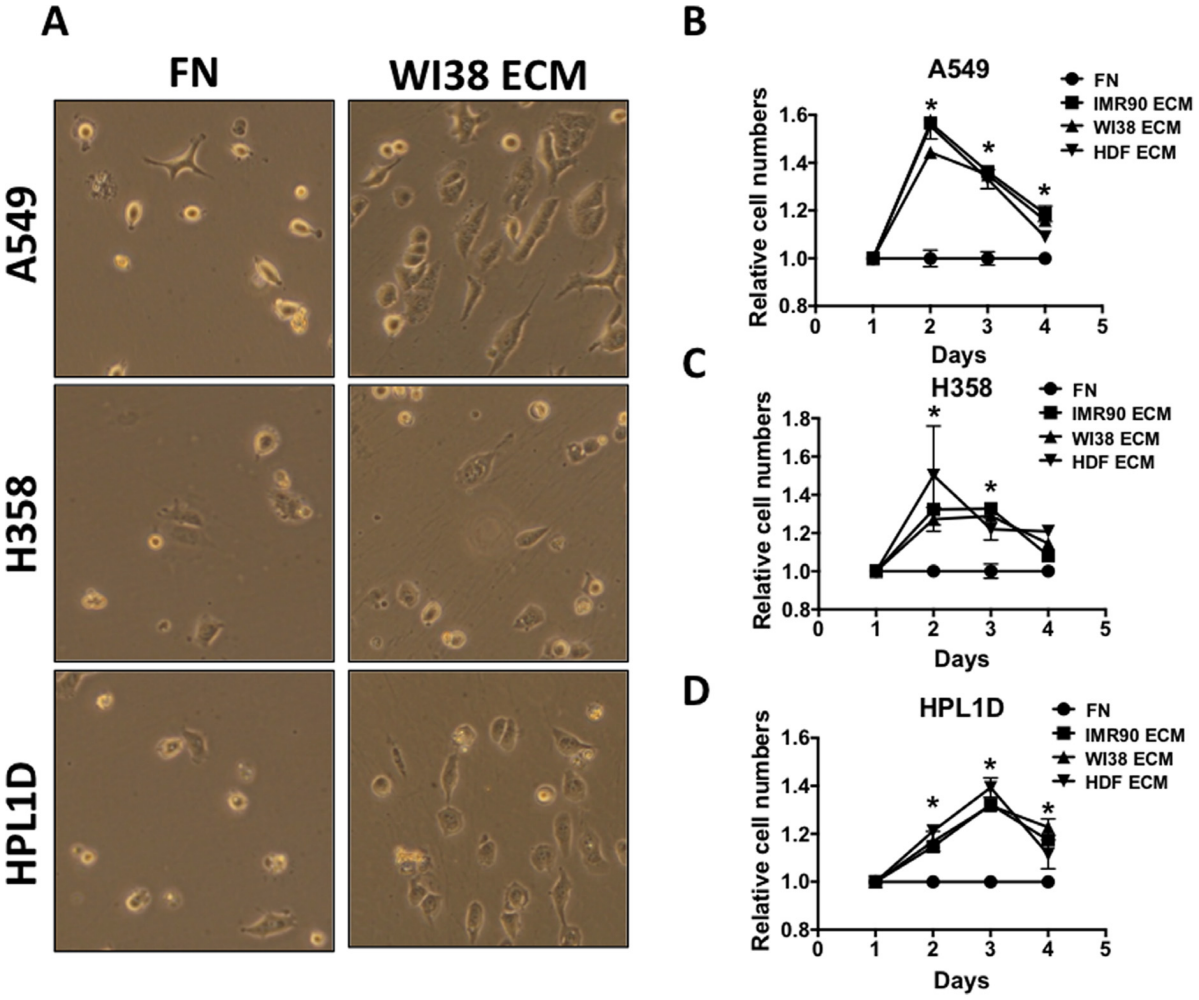
Fibroblast-derived ECM Protects Lung Cancer Cell Lines from Serum Deprivation. (A) A549, H358, and HPL1D cells were grown on fibronectin (FN) or on WI38 ECM in serum-free media for 48 hours and then photographed. (B) Relative cell numbers were quantified for A549 cells (B), H358 cells (C) and HPL1D cells (D) using Alamar Blue every 24 hours after cells were put into serum free media. By the fourth day in serum free media, basically all cells were dead.

Serum deprivation induces cellular apoptosis. However, little is known about how the ECM affects serum dependability [11]. We hypothesized that proteins and growth factors present in the fibroblast-derived ECM can sustain lung cancer cells in an otherwise nutrient-free environment. Observation by phase contrast microscopy revealed that 48 hours after the removal of serum from the media, there were more A549 cells on ECM compared to fibronectin coated dishes, suggesting that the ECM supports cell adhesion and survival in the absence of serum (Fig 5A). To determine the differences between relative cell numbers on ECM and fibronectin, Alamar Blue™ metabolism was monitored daily. All lung cell lines cultured in serum-free conditions on human lung and human dermal fibroblast-derived ECM showed an increase in the number of relative viable cells as compared to the same cell lines grown on fibronectin coated plates (Fig 5B, 5C and 5D). However, the ECM was not able to sustain cell viability in the absence of serum beyond four days. Perhaps this is due to degradation of the ECM and potential growth factors by the lung cancer cell lines. These results suggest that the ECM is capable of providing a cell-survival stimulus when the plethora of growth factors and nutrients available in FBS are absent. This combination of these observations (ECM, hypoxia and nutrient deprivation) might be important in understanding how lung adenocarcinoma cells survive in the inner-most core of a solid tumor, where nutrients, as well as oxygen, from the blood supply are scarce.

### Fibroblast-Derived Matrices Change mRNA levels of Various Genes in Lung Cancer Cells

**Fig 6:**
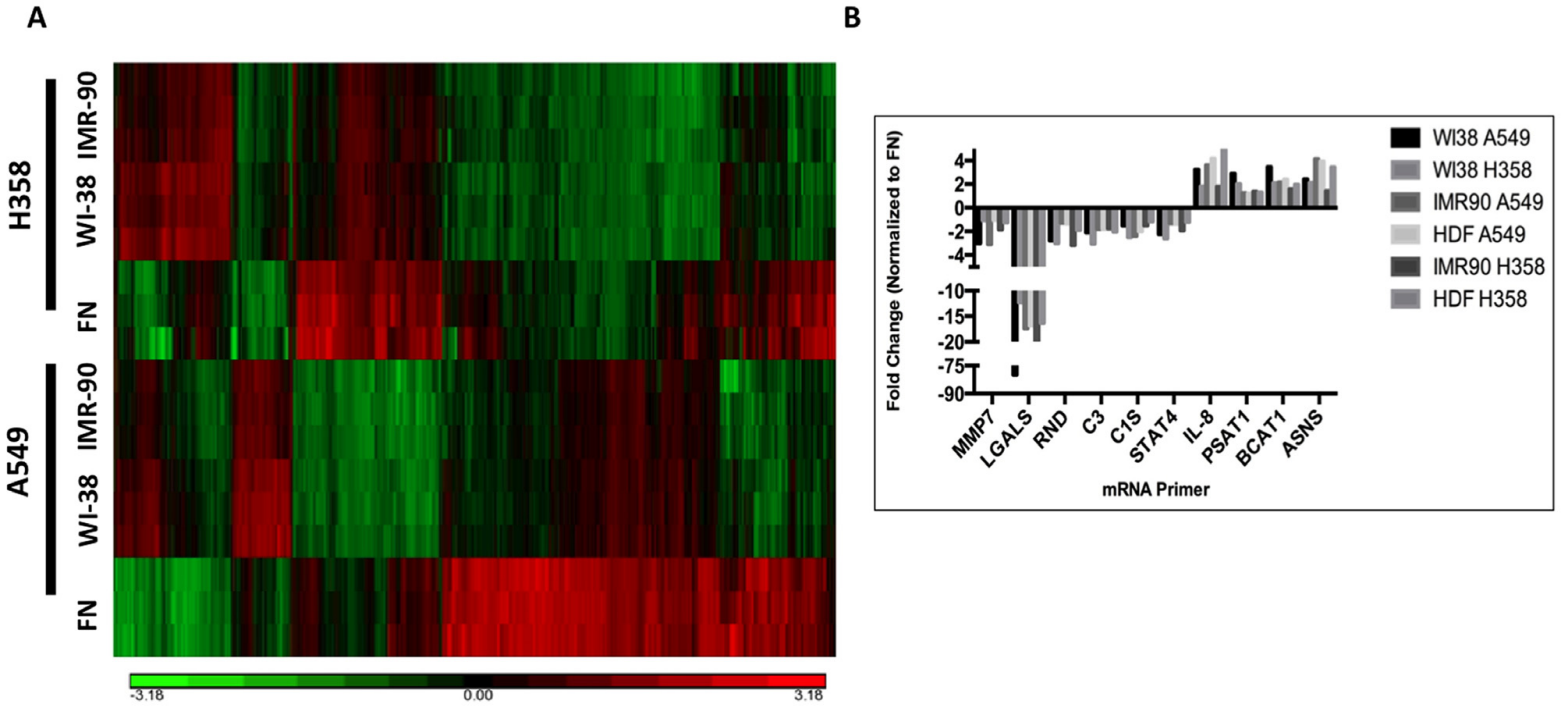
Fibroblast-derived ECM alters mRNA profile of A549 and H358 Cells. Heat map significantly changed genes from microarray (fold-change >1.5 and p-value ≤ 0.05). Columns represent individual gene probes; rows are the different samples and each row represents one of the biological triplicates ran on the microarray. (B) Validation of microarray data. Representative genes were chosen for quantitative real-time qRT-PCR analysis. New biological triplicates were prepared, RNA extracted and converted to cDNA, and real-time qRT-PCR was performed. All samples tested were validated and all genes were changed in the same direction as the microarray, however some amplitudes of change were slightly different.

It is clear that human lung fibroblast-derived ECM alters many processes that influence cellular behaviors, including morphology, growth, and survival following serum-deprivation. It is possible that these altered cellular behaviors are due to an alteration in gene transcription by the fibroblast-derived ECM. Transcription regulation is an extremely important process that influences cellular phenotypes. Therefore, a microarray was used to determine the difference of messenger-RNA (mRNA) copy number in lung cancer cell lines were cultured on fibroblast-derived ECM. Lung cancer cells, A549 and H358, were cultured on human fibroblast-derived ECM for 48 hours or on fibronectin-coated plastic. Briefly, RNA was extracted, converted to DNA, labeled, hybridized, and scanned for hybridization quantification. Data was then analyzed and a heat map of significantly altered genes was generated (Fig 6A). It is clear by examining the heat map that there are many gene probes that differ in expression induced by fibroblast-derived ECM. In total, 182 probes, which corresponded to 114 genes, were shared between both cells lines on both fibroblast-derived ECM compared to fibronectin-coated dishes. Interestingly, 87 of these significant gene changes were down-regulated, and only 27 were upregulated. Many down-regulated genes encoded for proteins that constitute the extracellular matrix, suggesting that when cells are in contact with adequate ECM, they decrease transcription of genes that code for ECM proteins such as fibrinogen alpha, fibrinogen beta, collagen type 4, collagen type5, and villin. Also, MMP7, which is a protease that degrades collagen and fibronectin, is also down-regulated. The most down-regulated gene, LGALS2 belongs to a family of galectins, which interact with ECM proteins and can be deregulated in some cancers, including lung cancer [23]. Also, several genes that regulate the complement-mediated immunity pathway, such as C3, C1S, and bradykinin, are down-regulated when both lung cancer cell lines are cultured on WI38 and IMR90 ECM. Complement-mediated immunity pathways have been shown to be activated in lung cancer [24]. Galectin expression also affects complement activation [25]. Up-regulated genes include NT5E, IL-8, KCNMA1, BCAT1, ASNS, stanniocalcin-2, and PSAT1. IL-8 is known to promote angiogenesis, thus supporting previous research by Soucy et al, suggesting a role for ECM in angiogenesis [17,18, 26]. NT5E, also known as ecto-5’-nucleotidase, has been shown to be upregulated in lung and breast cancers where it decreases patient survival rates by attenuating immune responses and promoting environments for vascularization and metastasis [27]. These microarray data reveal the complex mechanisms that the ECM can regulate within lung cancer cells. Thus, these data provide strong evidence that fibroblast-derived ECM can potentiate the phenotype of lung cancer cell lines.

To validate the microarray results, primers were designed to bind the mRNA region of various hits from the microarray data. Separate samples were prepared and analyzed to confirm the results. Real-time qRT-PCR analysis (Table 1 and Fig 6B) confirmed the results obtained from the microarray. Real-time qRT-PCR data showed similar direction and fold-changes in mRNAs as the microarray. These data give confidence to the effects of the fibroblast-derived ECM on the transcription of genes in the lung cancer cells that were observed on the microarray.

### Fibroblast-Derived Matrices Induce Lung Cancer Cell Migration

**Fig 7:**
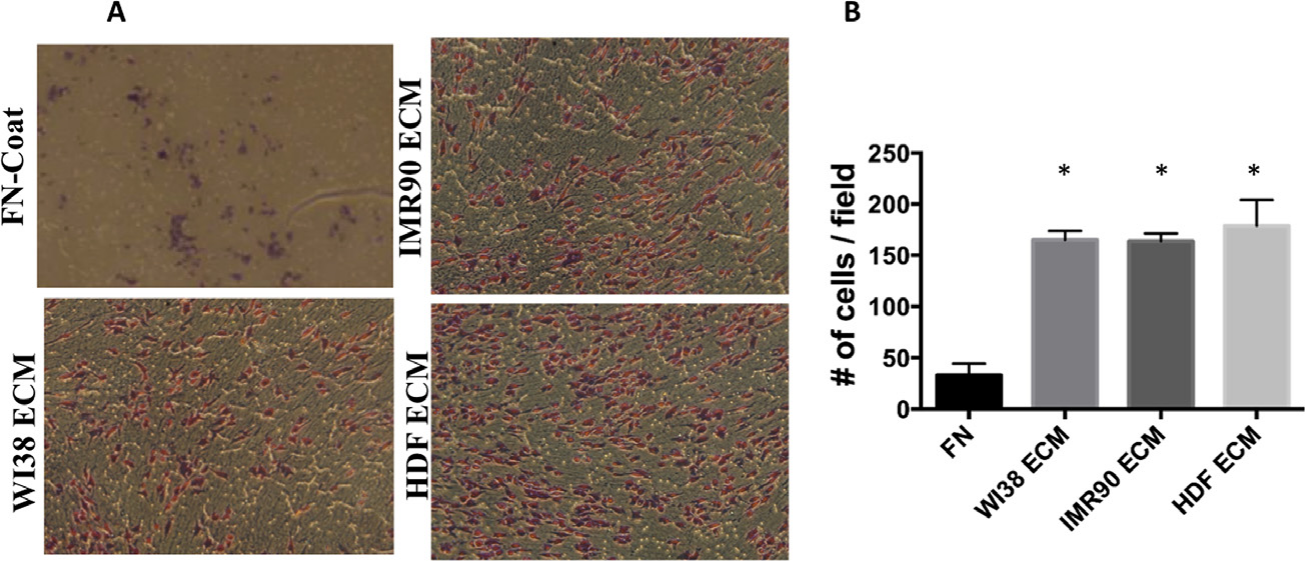
Fibroblast-derived ECM alter Cell Migration of Lung Cancer Cell Lines in a Trans-well Chamber. (A) The under side of a boyden chamber was coated with fibronectin and seeded with the indicated fibroblast cells. After eight days the ECM was prepared as described in the methods. A549 cells were then seeded inside the boyden chamber and 48 hours later the cells that migrated through the pores to the under-side containing the ECM were stained with H&Eand photographed. (B) Quantification of the cell migration. Stained trans-well membranes showing migrated cells were counted and the average migratory cells per field (4 fields) averaged over two experiments was calculated. *, p-value ≤ 0.05.

Changes in lung cancer gene expression and intracellular signaling are known to be play an important role in lung cancer cell migration and metastasis. It is unclear if the ECM can directly alter the ability of lung cancer cells to migrate. To determine if human fibroblast-derived ECM affects the migration of lung cancer cells, an experiment was designed employing a trans-well boyden chamber assay. Here, the under side of trans-well chambers were used as a substrate to grow fibroblasts and derive ECM. Once ECM was acquired, A549 cells were seeded inside the boyden chamber on the side of the porous filter opposite the ECM. It was hypothesized that human fibroblast-derived ECM would act as a chemoattractant that could recruit lung cancer cell lines to its side of the trans-well chamber. All fibroblast-derived ECM acted as a chemoattractant for A549 cells and induced them to migrate across the membrane (Fig 7A). Upon counting, three times the number of A549 cells migrated when fibroblast-derived ECM was present, as compared to fibronectin (Fig 7B). Cell migration is an important process that affects cancer progression. In cancer, local as well as distal ECM environments are equally as important in understanding cancer cell invasion and metastasis. Therefore, incorporation of fibroblast-derived ECM into trans-well migration could provide researchers with a better model to study cancer cell migration.

**Fig 8:**
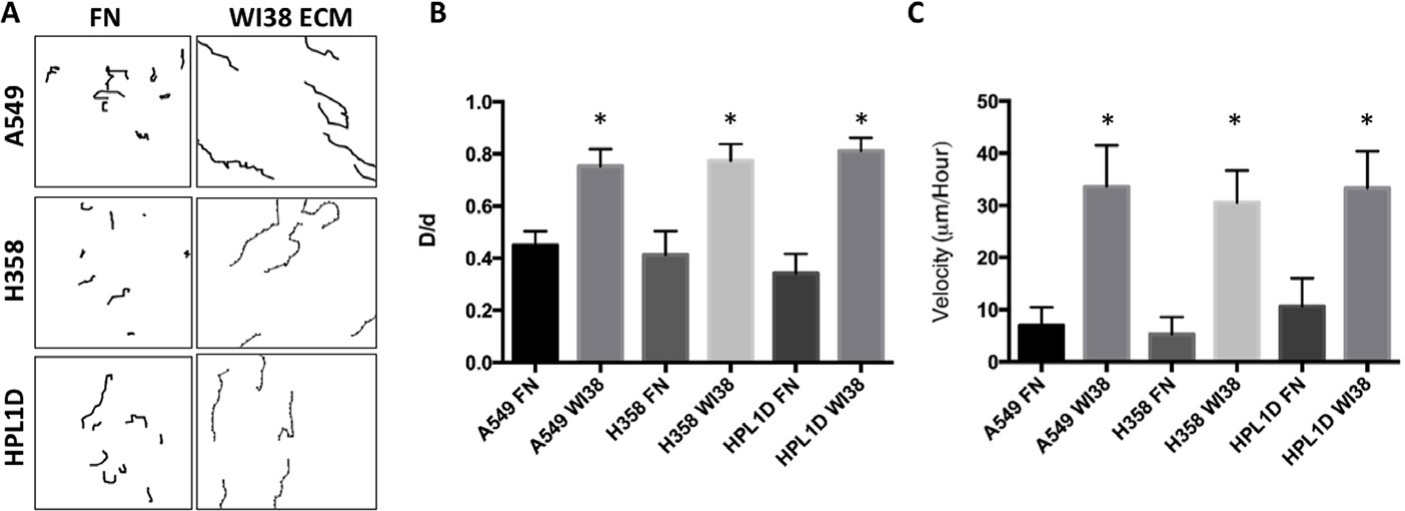
Fibroblast-derived ECM induce Directional Migration. (A) Cells were seeded on ECM or fibronectin and placed in a humidified temperature controlled chamber. Fields of cells were photographed every 10 minutes for approximately 8 hours. Resultant time-lapse tracks of A549, H358, and HPL1D on WI38 ECM were followed and indicated. (B) Directionality of the cells on different substrates was calculated. N = 7. (C) The average velocity of migrated cells was calculated from the time-lapse microscopy (μm/hour) n = 7. *, p-value ≤ 0.05.

It was postulated that cells grown on the 3D fibers of the ECM exhibit migration along the fibers, thus spreading the cells apart from each other. To test this type of migration, A549, H358 and HPL1D cells were seeded onto fibroblast-derived matrices for 24 hours and then the plates were transferred into a live cell microscopy chamber for time-lapse imaging (Fig 8). Time-lapse imaging shows that cells grown on fibroblast-derived matrix exhibited more migration than those grown on a fibronectin-coated dish. A549, H358, and HPL1D cells all migrated farther distances on fibroblast-derived ECM than on fibronectin (Fig 8A). To characterize this, directionality (D/d) was calculated to determine the linearity of the directional migration (Fig 8B). A549, H358, and HPL1D cell lines exhibited a more directional migration on fibroblast-derived ECM compared to cells grown on 2-dimension substrate such as fibronectin (Fig 8B). It appears that when the lung cancer cell lines interact with a fiber, they travel along that fiber only, rarely migrating towards adjacent fibers. This result will be important for future cell migration studies, encouraging researchers to use ECM that are highly aligned. In addition to directionality, the migration velocity was calculated by dividing the distance traveled by time it took to travel that distance. The velocity of the cells on fibroblast-derived ECM was significantly greater than the same cells on fibronectin (Fig 8C). Cell migration properties are highly adopted in lung cancer cells, which may allow them to invade accessible organs. These data indicate that the interaction between fibroblast-derived ECM and lung cancer cell lines induces a migratory phenotype. It is interesting that fibroblast-derived ECM alters EMT properties, thus suggesting that the ECM plays a role in EMT induction. Therefore, fibroblast-derived matrices should be used in the future to study ECM-induced EMT.

## Conclusion

The microenvironment surrounding tumors is a very heterogeneous mixture of cell types and extracellular matrix. The interplay between cancer cells and the microenvironment is therefore a complicated and tedious dynamic to study. In this work we attempt to simplify the system by only examining the interaction between lung epithelial cells and extracellular matrix secreted by human fibroblasts. We demonstrate that ECM can directly alter cell morphology, cell growth, proliferation, mRNA expression, functional protein expression, and migration properties of the lung-derived epithelial cell lines. It is clear that when cultured on human fibroblast-derived ECM, lung cancer cell lines have less activated MAPK activation as well as decreased mTOR and cell cycle regulating pathways. These alterations may be responsible for the decreased proliferation that was observed when cells are cultured on ECM. Also, lung cancer cells grown on ECM have altered expression of ECM protein-coding and complement-mediated immunity genes, thus affecting many downstream pathways. As discovered by western blot, lung cancer cell lines also develop an EMT-like phenotype, which could be responsible for their increased migration. Interestingly, ECM harvested from different fibroblast cell lines originating from different human tissues did not have a noticeable effect in this work. Perhaps a less harsh decellularization technique will leave behind a more unique ECM, thus allowing researchers to better mimic the natural ECM. T We feel that using this system much more closely resembles a situation that occurs *in vivo*, when compared to traditional cell culture. The main reason for this is because the interaction between a cancer cell and the solid tissue culture dish does not mimic any interaction that would ever occur within a tumor. In our system, the structure and components of a secreted ECM are in tact and the cancer cells are able to interact with this biological scaffold in a manner that would be more similar to what the cell might interact with *in vivo*. This work provides further evidence that the extracellular matrix has a strong effect on the phenotype of lung cancer cells *in vitro*. We suggest that future experiments in cell culture should incorporate the use of fibroblast-derived ECM in order to determine how relevant these interactions actually are and to determine if the signaling pathways altered on ECM more closely model the signaling pathways that occur within tumors.
